# Efficacy of laser monotherapy or non-surgical mechanical instrumentation in the management of untreated periodontitis patients. A systematic review and meta-analysis

**DOI:** 10.1007/s00784-020-03584-y

**Published:** 2020-11-10

**Authors:** Zhikai Lin, Franz J. Strauss, Niklaus P. Lang, Anton Sculean, Giovanni E. Salvi, Alexandra Stähli

**Affiliations:** 1 Department of Periodontology, School of Dental Medicine, University of Bern, Freiburgstrasse 7, 3010 Bern, Switzerland; 2 Department of Periodontology, Ninth People’s Hospital, Shanghai JiaoTong University School of Medicine, Shanghai, China; 3 Clinic of Reconstructive Dentistry, Center of Dental Medicine, University of Zurich, Zurich, Switzerland; 4 Department of Conservative Dentistry Faculty of Dentistry , University of Chile , Santiago, Chile

**Keywords:** Periodontitis, Laser, Laser monotherapy, Monotherapy, Non-surgical periodontal treatment, Systematic review

## Abstract

**Objective:**

To evaluate and compare the effects of laser monotherapy with non-surgical mechanical instrumentation alone in untreated periodontitis patients.

**Materials and methods:**

A focused question was formulated based on the Population, Intervention, Comparison, Outcome, and Study design criteria (PICOS): in patients with untreated periodontitis, does laser mono-therapy provide adjunctive effects on pocket probing depth (PPD) changes compared with non-surgical instrumentation alone? Both randomized controlled clinical trials (RCTs) and controlled clinical trials (CCTs) were included. The results of the meta-analyses are expressed as weighted mean differences (WMD) and reported according to the PRISMA guidelines.

**Results:**

The search yielded 1268 records, out of which 8 articles could be included. With respect to PPD changes, a meta-analysis including 5 articles (*n* = 148) failed to identify statistically significant differences in favor of laser monotherapy for PPD change (WMD = 0.14 mm; 95% CI: − 0.04/0.32; *z* = 1.51; *p* = 0.132) nor for clinical attachment level (CAL) (WMD = 0.04 mm; 95% CI: − 0.35/0.42; *z* = 0.19; *p* = 0.850). Data on cost-effectiveness are lacking. One study reported patient-related outcome measures (PROMS).

**Conclusions:**

In untreated periodontitis patients, laser monotherapy does not yield superior clinical benefits compared with non-surgical mechanical instrumentation alone.

**Clinical relevance:**

In untreated periodontitis patients, mechanical instrumentation with hand and/or ultrasonic instruments remains the standard of care.

## Introduction

Bacterial hard and soft deposits constitute the etiological agents in the initiation and progression of periodontitis [1]. Non-surgical mechanical instrumentation aims at removing the microbial biofilm and calcified deposits and to prepare a biologically acceptable surface that allows healing and regeneration [2, 3]. Subgingival calculus removal by hand and/or power-driven instruments is considered the standard of care [4]; it can, however, lead to excessive cementum removal [5], the creation of grooves, or leave calculus remnants. Anatomical critical sites often limit access to hand instruments. Furthermore, mechanical debridement results in a smear layer [6] containing bacteria, endotoxins, and contaminated cementum. Lasers have been incorporated in the treatment of periodontitis to reduce bacterial infection and bleeding, to improve access for complex anatomical structures, and to increase patient comfort and possibly stimulate the healing process. When using laser irradiation instead of mechanical non-surgical instrumentation, appropriate lasers that are able to remove plaque deposits and calculus have to be used.

The first use of a ruby laser for calculus removal was presented in 1965 [7]. The Nd:YAG laser, approved for hard tissues in 1999, was initially propagated for calculus removal. However, the wavelength of 1064 nm is mostly absorbed in dark tissues, and high irradiation output to remove calculus has been shown to cause thermal damage, such as carbonization [8] and chemical alterations of root proteins [9]. Of the lasers used in dentistry, the ER:YAG laser is largely absorbed in water with an absorbance 10 times higher than that of CO_2_ laser and 15000 to 20000 times higher than the one of Nd:YAG laser [10]. Ablation of both hard and soft tissues is possible, and due to its high absorption in water, Er:YAG lasers cause less damage to hard tissues containing small amounts of water [11-13]. Er:YAG lasers contain a yttrium aluminum garnet (YAG) crystal doped with erbium ions which generate a wavelength of 2936 nm. Wavelength is a major factor in the absorption of laser light by different biologic tissues. The laser light that is produced can be converted into kinetic energy in the form of shock waves, which then destroy the target tissue, in this case, calculus [14]. These shock waves are formed as a result of volumetric expansion which occurs when water evaporates. The calculus ablation primarily occurs through the evaporation of the water within the hard tissue itself. The resulting shock waves propagate through the calculus, spalling it from the underlying tissue—a process called laser spallation. Calculus contains a large volume of water and therefore absorbs the emission wavelength to a large extent. Secondly, the irrigation water serves as another evaporative medium where the explosive force of the vaporization of the thin film of water is transferred to the hard tissue, thus removing it [15]. These two effects combined increase the efficiency of the removal of the target tissue.

Recent systematic reviews have thoroughly documented the use of Er:YAG laser in non-surgical periodontal therapy [16]. It was reported that Er:YAG and Er,Cr:YSGG are able to sufficiently remove subgingival calculus. In a histologic study, the Er:YAG laser achieved plaque and calculus removal similar to hand instrumentation though leaving a rough surface morphology [17]. Similarly, the Er:YAG laser in vitro displayed increased loss of cementum and dentin [18] along with superficial thermal micro-changes compared with conventional scaling procedures [19]. Laser scaling further necessitated more time than ultrasonic scaling [19].

Despite reports of positive outcomes on the use of Er:YAG lasers in the management of untreated periodontitis, clinically relevant benefits for the patient need to be systematically appraised. Outcomes of a recent systematic review by our group focused on the combined nonsurgical therapy with laser and mechanical instrumentation and failed to indicate adjunctive benefits of laser application in the management of untreated periodontitis when compared with non-surgical mechanical instrumentation alone [20]. However, the potential benefits of laser monotherapy in the management of untreated periodontitis remain to be investigated. Therefore, the aim of the present systematic review was to assess and compare the effectiveness of laser monotherapy with that of non-surgical mechanical instrumentation alone in patients with untreated periodontitis.

## Material and methods

### Study registration

The protocol of this review was registered in the PROSPERO international prospective register of systematic reviews hosted by the National Institute for Health Research (NIHR), University of York, UK, Center for Reviews and Dissemination. The allocated number is CRD42020182626.

### Reporting format

The Preferred Reporting Items for Systematic Reviews and Meta-analyses were adopted throughout the process of the present systematic review [21–23].

### Population, Intervention, Comparison, Outcomes, and Study design

#### Population

Patients with untreated periodontitis

#### Intervention

Laser application alone

#### Comparison

Non-surgical mechanical instrumentation by means of hand and/or power-driven instrumentation alone

#### Outcome measures

##### Primary outcome

Change in pocket probing depths (PPD)

##### Secondary outcomes

Change in clinical attachment levels [24] (CAL)

Residual PPD

Change in Bleeding on Probing (BoP)

Change in plaque index (PlI)

Change in gingival crevicular fluid (GCF) biomarker levels

Microbiological change in subgingival plaque

Patient-reported outcome measures (PROMs)

##### Study design

The following study designs were considered:

- Randomized controlled clinical trials (RCTs)
- Prospective placebo-controlled clinical trials (CCTs)
- Studies with split-mouth and parallel-arm design

### Focused question

The following focused question was adapted using the Population, Intervention, Comparison, Outcomes, and Study design (PICOS) criteria [25]:

In patients with untreated periodontitis, how does laser monotherapy compare with non-surgical mechanical instrumentation alone in terms of PPD changes?

### Search strategy

#### Electronic search

A comprehensive and systematic electronic search of MEDLINE via PubMed, Scopus, and Cochrane Central Register of Controlled Trials (CENTRAL) databases was conducted for studies in humans published in English up to February 29, 2020. Language was limited to English.

The following search terms were used:

##### PubMed search terms

For the search in the PubMed library, combinations of controlled terms (MeSH) and keywords were used whenever possible:

(“periodontal diseases” [MeSH Terms] OR “periodontitis” [MeSH Terms]) AND (“laser” [All Fields]) AND (“non-surgical” [All Fields] OR “non surgical” [All Fields] OR “scaling” [All Fields] OR “root planing”[All Fields] OR “root planning”[All Fields] or “debridement”[All Fields] OR “conventional periodontal therapy” [All Fields])

##### Scopus search terms

(KEY (“periodontal diseases” OR “periodontitis”)) AND (TITLE-ABS-KEY (“laser”)) AND (TITLE-ABS-KEY (“non-surgical” OR “non-surgical” OR “scaling” OR “root planing” OR “root planning” OR “debridement” OR “conventional periodontal therapy”))

##### Cochrane database for randomized controlled trials search terms

(MeSH descriptor: [Periodontitis] explode all trees OR MeSH descriptor: [Periodontal Diseases] explode all trees) AND (All text (“laser”)) AND (All text (“non-surgical” OR “non-surgical” OR “scaling” OR “root planing” OR “root planning” OR “debridement” OR “conventional periodontal therapy”))

#### Manual search

A manual search of the reference lists of relevant reviews and systematic reviews on the topics as well as of the reference lists of the included full-text articles was performed.

### Inclusion criteria

The inclusion criteria were:

- Laser therapy alone as one of the treatment groups and non-surgical mechanical instrumentation as control group.
- Follow-up of at least 6 months with clinical examination.
- At least 20 patients per treatment arm at 6-month follow-up.
- At least 20 patients at 6-month follow-up for studies with split-mouth design.
- Non-surgical instrumentation by means of hand and/or power-driven instruments.
- For meta-analyses: RCTs/CCTs reporting a single session of non-surgical mechanical instrumentation alone or laser monotherapy and PPD/CAL changes at the 6-month follow-up.

### Exclusion criteria

The exclusion criteria were:

- Studies including patients with treated periodontitis or in the course of supportive periodontal therapy (SPT) or referred patients with pre-treated periodontitis.
- Studies including a combination of laser and SPT or laser as adjunctive therapy.
- Abstracts
- Letters to editors
- Narrative reviews
- Case reports or case series
- Insufficient/unclear information not allowing data extraction
- No author response to inquiry e-mail for data clarification

#### Screening

Literature screening was performed independently by two reviewers (A.S. and Z.L.). Discrepancies were solved by discussion among authors. Cohen’s Kappa score was calculated to measure the agreement between the reviewing authors. The reviewers independently performed the search and screening process.

### Data extraction

Data addressing the primary and secondary outcome measures were extracted in duplicate by two independent reviewers (Z.L. and A.S.) for qualitative and quantitative analysis from the selected articles fulfilling the inclusion criteria.

### Quality assessment

The criteria used to evaluate the quality of the selected controlled trials were adopted from the checklist of the Cochrane Center and the CONSORT (Consolidated Standards of Reporting Trials) statement, providing guidelines for the following parameters: (a) sequence generation; (b) allocation concealment method; (c) masking of the examiner; (d) address of incomplete outcome data; and (e) free of selective outcome reporting.

The degree of bias was categorized as low risk if all the criteria were met, moderate risk when only one criterion was missing, and high risk if two or more criteria were missing. Potential impact of risk of bias for sample size calculation, patient selection, and reporting was considered for each selected study.

### Data analysis

Changes in periodontal parameters between baseline and the follow-up period were calculated using the following formulae;

1. If PPD or CAL pre- and post-intervention mean difference was not directly reported in the studies, then it was calculated according to the following formula, ΔPD = PD_2_ − PD_1_, where ΔPD stands for the reduction of probing depth; PD_2_ stands for the post-treatment probing depth value, while PD_1_ is the pre-treatment probing depth value.
2. If the standard deviation of the pre- and post-intervention mean difference was not reported in the studies, then it was calculated according to the following formula: SD = √ (SD_1_^2^ + SD_2_^2^ − 2*r* × SD_1_ × SD_2_); the coefficient r was calculated according to [26].

Results documenting PPD and CAL changes were extracted or calculated from RCTs and used to evaluate the effect of laser monotherapy compared with non-surgical mechanical instrumentation in patients with untreated periodontitis.

The results for continuous data such as changes in PPD (primary outcome) and CAL (secondary outcome) at the 6-month follow-up were measured with weighted mean differences (WMD) and 95% confidence intervals (CIs). A random-effect model was used to calculate the pooled WMDs, and *z* test was applied to determine the statistical significance for pooled WMDs. Forest plots were used to illustrate the outcomes of the meta-analyses. The statistical heterogeneity among studies was explored by the *I*^2^ index [27]. If *I*^2^ was found larger than 75%, then the risk of heterogeneity was high.

Statistical significance was set to *p* < 0.05.

## Results

### Search

**Fig. 1:**
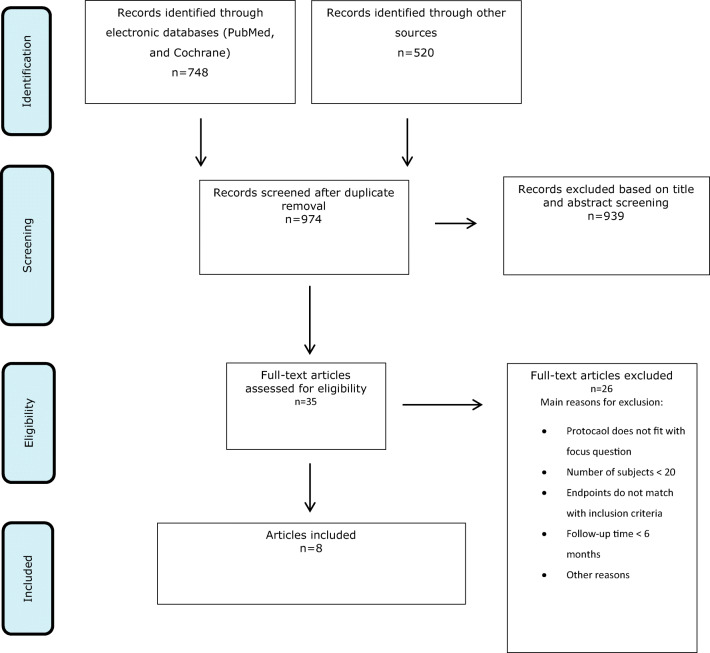
PRISMA flow chart depicting the selection process

A total of 1268 records were identified through the electronic search. After removal of 294 duplicates, 974 records remained for title and abstract screening. Based on abstract screening, another 939 articles were excluded. No citations from the manual search and the gray literature search were identified (Fig. 1).

A total of 35 articles remained for full-text evaluation. Following exclusion of 26 articles based on full-text analysis (Table 1), 8 articles remained to be included.

**Table 1:** Studies excluded based on full-text analysis and reasons for exclusion

First author (year of publication)	Reason for exclusion
Alzoman and Diab (2016) [28]	1
Ambrosini et al. (2005) [29]	2
Amid et al. (2017) [30]	3
Andersen et al. (2007) [31]	3
Badran et al. (2012) [32]	1
Bocher et al. (2019) [33]	3
Castro et al. (2019) [34]	3
Ciurescu et al. (2019) [35]	3
Derdilopoulou et al. (2007) [36]	3
Everett et al. (2017) [37]	3
Foroutan et al. (2013) [38]	3
Ge et al. (2017) [39]	2 and 3
Gianelli et al. (2012) [40]	3
Gomez et al. (2009) [41]	3
Harmouche et al. (2019) [42]	3
Jensen et al. (2010) [43]	1
Krohn-Dale et al. (2012) [44]	1
Lopes et al. (2008) [45]	2
Malali et al. (2012) [46]	2
Miyazaki et al. (2003) [47]	1
Moritz et al. (1997) [48]	1
Moritz et al. (1998) [49]	3
Noro Filho et al. (2012) [50]	3
Saglam et al. (2017) [51]	2
Soo et al. (2012) [52]	3
Yilmaz et al. (2002) [53]	3

1, number of subjects < 20; 2, follow-up time < 6 months; 3, study protocol does not match with stated focused question; 4, endpoints do not match with stated inclusion criteria; 5, no data at 6-month follow-up; 6, other reasons (e.g., follow-up time unknown); *no author response to inquiry e-mail for data clarification

An inter-examiner Cohen’s kappa score was calculated according to the results from title and abstract screening. The kappa score between the two examiners was 0.798.

### Laser monotherapy

#### Description of included studies

The characteristics of the 8 articles (7 studies) evaluating laser monotherapy are summarized in Table 2 [54–61].

**Table 2:** Characteristics of studies on laser monotherapy compared with non-surgical instrumentation

First author (year)	Study type design n center industrial funding calibration	Patient characteristics n patients (*n* female) mean age ± SD (range) periodontal diagnosis smoking status *n*-treated teeth/sites per treatment arm	Laser laser type (product name) material of tip (diameter) application time/site	Physical data laser power laser energy irradiation time wavelength laser intensity laser density	Treatment	Follow-up time points treatment adverse effects
Schwarz (2001)	RCT split-mouth single-center n.r. examiner calibrated	20 patients (14 female) 54 (28-79) moderate to advanced periodontal disease n.r. 34 maxillary and 21 mandibular single and multirooted teeth/330 sites	Er:YAG laser (KEYII, KaVo, Biberach, Germany) Fiber tip 0.5 × 1.65 (136 mJ/pulse) and 0.5 × 1.1 (114 mJ/pulse) 5 min for single-rooted, 10 min for multi-rooted teeth	n.r. 160 mJ/pulse, 10 Hz n.r. 2.94 μm n.r. n.r.	test group: Er:YAG laser control group: SRP (hand)	3 and 6 months OHI and supragingival scaling at 3, 6 months none
Schwarz (2003)	RCT split-mouth single-center n.r. examiner calibrated	20 patients (14 female) n.r. moderate to advanced periodontal disease n.r. 34 maxillary and 21 mandibular single and multirooted teeth/330 sites	Er:YAG laser (KEYII, KaVo, Biberach, Germany) Fiber tip 0.5 × 1.65 (136 mJ/pulse) and 0.5 × 1.1 (114 mJ/pulse) 5 min for single-rooted, 10 min for multi-rooted teeth	n.r. 160 mJ/pulse, 10 Hz n.r. 2.94 μm n.r. n.r.	test group: Er:YAG laser control group: SRP (hand)	12 and 24 months OHI and supragingival scaling at 3, 6,12, 18, and 24 months none
Sculean (2004)	RCT split-mouth single-center n.r. examiner calibrated	20 patients (n.r.) 51 (29–62) Moderate to advanced periodontal disease n.r. ErYAG: single-rooted: 407 sites multi-rooted: 269 sites US: single-rooted:383 sites multi-rooted:247 sites	Er:YAG laser (KEY3, KaVo, Biberach, Germany) with a calculus detection system with fluorescence induced by 655 nm InGaAsP diode laser Fiber tip 0.5 x 1.65 (136 mJ/pulse) and 0.5 x 1.1 (114 mJ/pulse) 5 min for single-rooted, 9 min for multi-rooted teeth	n.r. 160 mJ/pulse, 10 Hz n.r. 2.94 μm n.r. n.r.	test group: Er:YAG with a calculus detection system with fluorescence induced by 655 nm InGaAsP diode laser control group: SRP (ultrasonic)	3 and 6 months OHI and supragingival scaling at 2, 4, 6, 8, 10, 12, 16, 20, and 24 weeks none
Crespi (2007)	RCT split-mouth single-center none examiner calibrated	25 patients (15 female) 53 (37-65) Moderate to advanced ChP n.r. 65 single-rooted, 35 multi-rooted teeth / 600 sites	Er:YAG laser (HOYA ConBio, Fremont, CA, USA) Chisel shaped tip with 400 μm in diameter 5 min for single and 9 min for multi-rooted teeth	n.r. 160 μJ/pulse, 10 Hz n.r. 2.94 μm n.r. 94 J/cm^2^	test group: Er:YAG laser control group: SRP (ultrasonic)	3, 12 and 24 months OHI and supragingival scaling at 6, 12, 18, and 24 months n.r.
Kamma (2009)	RCT split-mouth single-center none examiner calibrated	30 patients (16 female) 41.8 ± 6.2 AgP 18 smokers, 12 non-smokers One quadrant	diode laser (SmilePro980™; Biolitec, Jena, Germany) Flexible glass fiber optic guide with a 300-μm spot diameter	2 W n.r. 30 sec/site 980 nm 2830 W/cm^2^ 94.3 J/cm^2^	test group: laser (alone) SRP(hand) + laser control group: SRP (hand) No treatment	Weeks: 2–12–24 n.r. n.r.
Rotundo (2010)	RCT split-mouth single-center none examiner calibrated	27 patients (18 female) 50.5 ± 11.7 ChP < 10 cig./day One quadrant	Er:YAG laser (Smart 2940 Plus, DEKA M.E.L.A. srl, Calenzano, Florence, Italy) Conic fiber tip of 0.5 mm diameter n.r.	n.r. 150 mJ/pulse, 10 Hz n.r. 2.94 μm n.r. n.r.	test groups: Er:YAG(alone) SRP (hand + ultrasonic) + Er:YAG control group: SRP (hand and ultrasonic)	Weeks: 1–4–12–24 OHI and supragingival scaling at 3 and 6 months; 5 peridontal abscesses, 2 teeth lost. 1 patient with fever
Wang (2017)	RCT split-mouth single-center none examiner calibrated	27 patients (14 female) 43.6 ± 8.7 (28-56) ChP Non-smokers half-mouth 304 teeth/1087 sites in the test group and 308 teeth/1122 sites in the SRP group PPD ≥ 4 mm	Er:YAG laser (LAEDL001.1, Doctor Smile,Italy) Chisel-shaped fiber tip (1.1 mm × 0.5 mm)	Up to 6 W 160 mJ/pulse 10 Hz n.r. 2940 nm n.r. n.r.	test group: Er:YAG laser control group: SRP (hand an ultrasonic)	6 weeks–3–6 months OHI and supragingival debridement (ultrasound cleaning and polishing); none
Grzech-Lesniak (2018)	RCT parallel single-center none n.r.	60 patients (34 female) mean age 49.3 ± 11.6 (31-79) ChP Non-smokers 65 teeth in the SRP group, 65 teeth in the Er group, and 63 teeth in the NdErNd group	Er:YAG laser (LightWalker, Fotona d.o.o., Slovenia) fiber tip diameter 400 μm Nd:YAG laser (LightWalk, Fotona d.o.o., Slovenia) fiber tip diameter 300 μm Er:YAG n.r. Nd:YAG 10-30 sec/tooth	Er:YAG: 2.5 W, 20 Hz 40 mJ/pulse 100 μs Nd:YAG 100 μs Er:YAG 2940 nm Nd:YAG 1064 nm n.r. n.r	test groups: Er:YAG Er:YAG and Nd:YAG For Er:YAG and Nd YAG group additional two sessions of Nd:YAG treatmets in 7-day interval control group: SRP (hand)	Monthly OHI and supragingival scaling n.r.

#### Study design

Two articles included two experimental and two control groups [58, 59]. One article included two experimental groups and one control group [61] while the remaining 5 articles included one experimental and one control group, respectively.

One article reported on a parallel arm design [61] while the remaining 7 articles reported on a split-mouth design. One article [55] published the 2-year follow-up data of a previous article [54]. Two articles reported 2-year follow-up data [55, 57] while the follow-up of the other 6 articles was 6 months.

The total number of patients treated was 209 of whom 149 were included in studies with a split-mouth and 60 in studies with a parallel arm design, respectively.

#### Study samples

Sample sizes of the included studies varied from 20 to 60 patients. The age of the included patients ranged from 28 to 79 years and the mean age from 41.8 to 53 years. The percentage of females ranged from 51.8 to 70.0% and of males from 30.0 to 48.9%, respectively. Smokers were included in 2 studies [58, 59], excluded in another two studies [60, 61], whereas the remaining studies did not report on tobacco consumption. One study reported on patients diagnosed with aggressive periodontitis [58], and 6 studies on patients diagnosed with chronic periodontitis [54–57, 59–61].

All studies were performed in single centers. Only one study [58] was conducted in a private dental clinic while the remaining 6 studies were conducted in a university setting.

#### Intervention/comparison

Three different types of laser were used in the 7 included studies; 2 different kinds of lasers were used in one study [61]. Diode laser was used in 1 study [58], Er:YAG laser in 6 studies [54–57, 59–61], and Er:YAG laser and Nd:YAG laser in 1 study [61].

In all studies, non-surgical mechanical instrumentation and laser monotherapy were reported to be performed in one session except for the combined Er:YAG/Nd:YAG laser treatment group in one study [61]. In that group, two additional sessions of Nd:YAG treatments were applied after Er:YAG laser treatment. The physical parameters of the lasers are summarized in Table 2.

For non-surgical mechanical instrumentation, 3 studies reported using hand instruments only [54, 55, 58, 61] while 2 studies reported using power-driven devices only [56, 57] and 2 studies mentioned using a combination of hand instruments and power-driven devices [59, 60].

As far as the application of local anesthesia was concerned, 3 studies reported the use of local anesthesia [56, 58, 61], in 1 study local anesthesia was reported to be delivered if needed [59], and the remaining 2 studies did not provide any information related to the use of local anesthesia.

#### Outcomes

Clinical outcome parameters of the 8 articles (7 studies) evaluating laser as monotherapy are shown in Table 3. In order to perform meta-analyses on the primary (i.e., PPD change) and secondary outcome measure (i.e., CAL change), 5 articles including the 6-month PPD and CAL changes and reporting on non-surgical mechanical instrumentation or laser monotherapy in one session were selected [54, 56, 59–61].

**Table 3:** Clinical outcome parameters of studies using laser monotherapy compared with non-surgical mechanical instrumentation alone

First author (year)	Group	Time point	PPD (mm)	PPD change (mm)	CAL (mm)	CAL change (mm)	GR (mm)/GR change^^^ (mm)	BOP (%)	BOP change (%)	GI	PI (%) or PLI/PLI change^^^
Schwarz (2001)	Test	Baseline	4.9 ± 0.7	-	6.3 ± 1.1	-	1.4 ± 0.8	56	-	1.9 ± 0.6	1.0 ± 0.6
		6 months	2.9 ± 0.6*	2.0 ± 0.64^#^	4.4 ± 1.0*	1.9 ± 0.86^#^	1.5 ± 0.7*	13*	-	0.3 ± 0.6	0.7 ± 0.4
	Control	Baseline	5.0 ± 0.6	-	6.5 ± 1.0	-	1.5 ± 0.8	52	-	1.9 ± 0.6	1.0 ± 0.6
		6 months	3.4 ± 0.7	1.6 ± 0.64^#^	5.5 ± 1.0	1.0 ± 0.81^#^	2.0 ± 0.8	23	-	0.4 ± 0.8	0.7 ± 0.5
Schwarz (2003)	Test	Baseline	4.9 ± 0.7	-	6.3 ± 1.1		1.4 ± 0.8	56	-		1.0 ± 0.6
		12 months	3.0 ± 0.8*	1.9	4.5 ± 1.3*		1.5 ± 0.7*	14	-		0.6 ± 0.4
		24 months	3.3 ± 0.9*	1.6	4.9 ± 1.0*	-0.1^1^, 1.1^2^, 3.3^3^	1.6 ± 0.7*	20			1.3 ± 0.6
	Control	Baseline	5.0 ± 0.6	-	6.5 ± 1.0		1.5 ± 0.8	52	-		1.0 ± 0.6
		12 months	3.5 ± 1.3	1.5	5.6 ± 1.4		2.1 ± 0.7	26	-		0.7 ± 0.5
		24 months	3.7 ± 0.7	1.3	5.8 ± 1.0	-0.7^1^, 0.8^2^,1.9^3^	2.1 ± 0.7	28			1.2 ± 0.6
Sculean (2004)	Test	Baseline	5.28 ± 0.6	-	6.78 ± 1.03	1.11 ± 0.59	1.49 ± 0.75	40	-		0.78 ± 0.15
		6 months	-	1.52 ± 0.57	-	0.6 ± 0.4^2^, 1.8 ± 1.7^3^	-0.41 ± 0.16^^^	-	23		0.02 ± 0.13^^^
	Control	Baseline	5.33 ± 0.6	-	6.74 ± 0.85	1.11 ± 0.46	1.41 ± 0.6	46	-		0.81 ± 0.16
		6 months	-	1.57 ± 0.46	-	0.6 ± 0.5^2^, 1.9 ± 1.7^3^	-0.46 ± 0.20^^^	-	31		0 ± 0.12^^^
Crespi (2007)	Test	Baseline^2^	5.49 ± 0.27	-	6.27 ± 0.51	-	-	-	-	1.75 ± 0.58	1.05 ± 0.51
		12 months^2^	2.60 ± 0.37*	2.89	3.32 ± 0.64*	2.95	-	-	-	0.64 ± 0.42	1.26 ± 0.57
		24 months^2^	2.61 ± 0.54*	2.88	3.35 ± 0.91*	2.92	-	-		1.09 ± 0.61	1.29 ± 0.48
	Control	Baseline^2^	5.12 ± 0.39	-	6.18 ± 0.42	-	-	-	-	1.75 ± 0.58	1.05 ± 0.51
		12 months^2^	4.02 ± 0.65	1.10	4.89 ± 0.55	1.29	-	-	-	0.63 ± 0.35	1.27 ± 0.64
		24 months^2^	4.12 ± 0.74	1.00	4.86 ± 0.52	1.32	-	-	-	1.01 ± 0.76	1.28 ± 0.65
	Test	Baseline^3^	7.92 ± 0.78	-	8.41 ± 0.47	-	-	-	-	1.75 ± 0.58	1.05 ± 0.51
		12 months^3^	3.11 ± 0.41*	4.01	4.40 ± 1.01*	5.10	-	-	-	0.64 ± 0.42	1.26 ± 0.57
		24 months^3^	3.05 ± 0.53*	4.87	3.38 ± 0.79*	5.03	-	-		1.09 ± 0.61	1.29 ± 0.48
	Control	Baseline^3^	7.13 ± 0.53	-	8.35 ± 0.33	-	-	-	-	1.75 ± 0.58	1.05 ± 0.51
		12 months^3^	4.82 ± 0.37	2.59	6.33 ± 0.61	2.02	-	-	-	0.63 ± 0.35	1.27 ± 0.64
		24 months^3^	4.85 ± 0.64	2.28	6.34 ± 0.92	2.01	-	-	-	1.01 ± 0.76	1.28 ± 0.65
Kamma (2009)	Test	Baseline	5.93 ± 1.16	-	6.87 ± 1.60	-	-	50.7	-	-	83.7
		6 months	3.93 ± 1.31	2.00 ± 1.20^#^	4.93 ± 1.62	1.94 ± 1.31^#^	-	31.6	-	-	23.2
	Control	Baseline	6.47 ± 1.35	-	7.07 ± 1.58	-	-	54.1	-	-	81.6
		6 months	4.13 ± 1.6	2.34 ± 1.45^#^	5.20 ± 1.66	1.87 ± 1.32^#^	-	32.6	-	-	25.8
Rotundo (2010)	Test	Baseline	5.2 ± 1.2	-	6.2 ± 1.8	-	0.9 ± 1.2	75	-	-	64
		6 months	4.5 ± 1.9	0.7 ± 1.6	6.0 ± 2.4	0.2 ± 1.9	1.5 ± 1.4	58	-	-	42
	Control	Baseline	5.2 ± 1.2	-	6.1 ± 1.6	-	0.8 ± 1.1	73	-	-	68
		6 months	4.3 ± 1.7	1.0 ± 1.5	5.6 ± 2.0	0.5 ± 1.8	1.3 ± 1.3	57	-	-	48
Wang (2017)	Test	Baseline^2^	4.73 ± 0.63	-	5.03 ± 0.53	-	-	-	-	3.19 ± 0.37	2.48 ± 0.78
		6 months^2^	3.55 ± 0.55	1.18	4.24 ± 0.74	0.79	-	-	-	1.87 ± 0.84	2.38 ± 0.74
		Baseline^3^	7.34 ± 0.53	-	7.69 ± 1.05	-	-	-	-	4.01 ± 0.39	2.59 ± 0.75
		6 months^3^	4.96 ± 1.30	2.38	5.63 ± 1.50	2.06	-	-	-	2.41 ± 0.91	2.47 ± 0.75
	Control	Baseline^2^	4.78 ± 0.79	-	5.03 ± 0.46	-	-	-	-	3.24 ± 0.25	2.50 ± 0.81
		6 months^2^	3.38 ± 0.70*	1.4	4.24 ± 0.74	0.79	-	-	-	1.81 ± 0.64	2.30 ± 0.75
		Baseline^3^	7.29 ± 0.34	-	7.64 ± 0.90	-	-	-	-	4.03 ± 0.19	2.56 ± 1.79
		6 months^3^	5.23 ± 0.93	2.06	5.66 ± 1.35	1.98	-	-	-	2.33 ± 1.00	2.48 ± 0.78
Grezch (2018)	Test1	Baseline	2.53 ± 0.15	-	3.45 ± 0.19	-	0.91 ± 0.17	18.7 ± 1.20	-	-	10.3 ± 1.27
	Er-YAG	6 months	2.01 ± 0.11	0.52 ± 0.13^#^	3.15 ± 0.21	0.47 ± 0.10	1.15 ± 0.16	10.6 ± 1.79*	-	-	6.15 ± 1.39*
	Test2 NdErNd	Baseline	2.43 ± 0.19	-	3.19 ± 0.19	-	0.75 ± 0.11	15.85 ± 1.42	-	-	9.16 ± 1.11
		6 months	1.73 ± 0.12*	0.70 ± 0.16^#^	2.72 ± 0.18*	0.69 ± 0.13	1.02 ± 0.12	7.4 ± 1.04*	-	-	4.05 ± 0.42*
	Control	Baseline	2.31 ± 0.16	-	3.30 ± 0.21	-	0.99 ± 0.14	17.57 ± 1.58		-	9.89 ± 1.45
		6 months	2.01 ± 0.17	0.30 ± 0.16^#^	3.05 ± 0.21	0.73 ± 0.10	1.04 ± 0.14	16.01 ± 1.41		-	8.10 ± 0.79

If not otherwise indicated, parameters are presented as means ± standard deviation

^1^For shallow sites (1–3 mm); ^2^for moderately deep sites (4–6 mm); ^3^for deep sites (> 7 mm); * statistically significant (*p* < 0.05); ^#^data calculated according to the formula in Cochrane workbook, using correlation coefficient calculated from the study of Rotundo 2010 (i.e., 0.53 for PPD change and 0.67 for CAL change)

Funnel plots are not reported to illustrate publication bias, based on the small number of studies in both meta-analyses (i.e., < 10).

#### Primary outcome: change in PPD

**Fig. 2:**
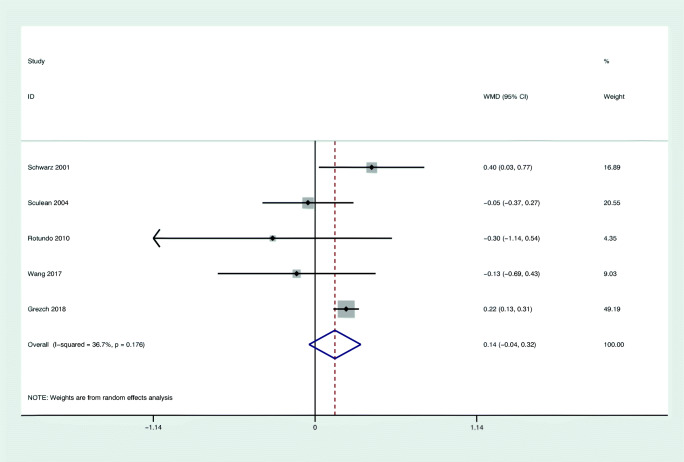
Forest plot of the weighted mean change in PPD at 6 months with laser as monotherapy compared to non-surgical mechanical instrumentation alone

Figure 2 shows the results of the meta-analysis for changes in PPD based on 5 studies evaluating 148 patients [54, 56, 59–61]. Two of 5 selected studies favored laser monotherapy [61, 54]; meanwhile, the rest 3 studies demonstrated slightly better improvement in the control groups than in test groups. No statistically significant difference (WMD = 0.14 mm; 95% CI: − 0.04/0.32; *z* = 1.51; *p* = 0.132) was found comparing the use of laser monotherapy with non-surgical mechanical instrumentation alone. The heterogeneity across the studies was low for PPD change (*I*^2^ = 36.7%).

Interestingly, PPD changes for sites with different initial PPD were compared between laser monotherapy and non-surgical mechanical instrumentation in 5 separate studies [54–57, 60, 61]. These studies grouped the sites with PPD of 5 and 6 mm and PPD of 7 mm or more as moderately deep sites and deep sites [54, 60], as shallow sites with 4–5 mm PPD and sites with > 5 mm PPDs [56] or as 4–6 mm and sites with > 6 mm [61]. At the 2-year follow-up, PPD showed more reduction in the Er:YAG laser group compared with the SRP group in both moderately deep and deep sites, respectively [55]. Furthermore, the difference was more significant in initially deep pockets than in shallow or moderate pockets.

#### Secondary outcomes

**Fig. 3:**
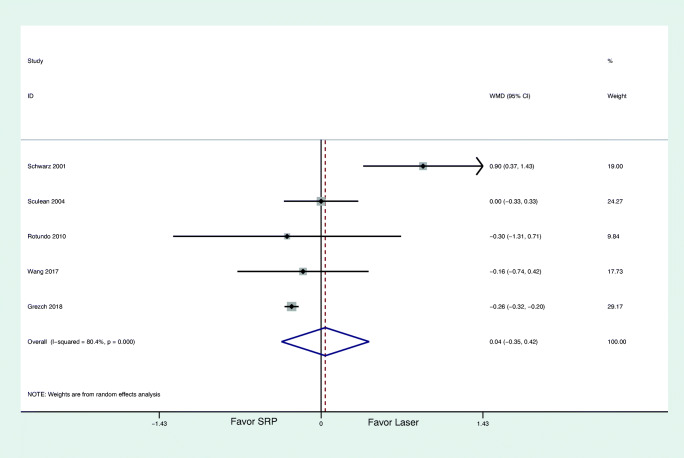
Forest plot of the weigthed mean change in CAL at 6 months with laser as monotherapy compared to non-surgical mechanical instrumentation alone

Figure 3 summarizes the results of the meta-analysis for changes in CAL based on 5 studies [54, 56, 59–61]. No statistically significant difference (WMD = 0.04 mm; 95% CI: − 0.35/0.42; *z* = 0.19; *p* = 0.850) was found comparing the use of laser monotherapy with non-surgical mechanical instrumentation alone. The heterogeneity across the studies was high for CAL change (*I*^2^ = 80.4%). Only one study favored the laser therapy [54], whereas the remaining 4 studies reported almost the same CAL gain between laser monotherapy and non-surgical mechanical instrumentation.

CAL changes within sites of different initial pocket depth were reported in 5 studies [54–57, 60, 61]. Two studies reported identical CAL changes for all of the sites between the two treatment modalities at the 6-month follow-up [56, 60]. While two studies with 2-year follow-up reported that initially deeper pockets (≥ 7 mm) showed the greatest changes in CAL, and moderately deep pockets exhibited moderate improvements, shallow sites (< 4 mm) showed the least amount of changes [55, 57]. In a more recent study, Grzech-Leśniak reported that both Er:YAG laser alone and in combination with Nd:YAG laser showed less CAL loss than the SRP group in shallow periodontal pockets < 4 mm, but all treatments reduced PPD and CAL significantly without differences between treatment modalities in deep periodontal pockets at 6 months [61]. For the moderately deep pockets, Er:YAG alone and SRP exhibited no statistically difference in CAL change, while the combined Nd:YAG and Er:YAG laser group significantly gained CAL.

Table 4 summarizes the studies reporting on all selected outcomes. A total of 6 studies reported residual PPD, 3 of them detected the statistical difference between laser monotherapy and SRP. Two studies reported less residual PPD in laser group at both 1 and 2-year follow-up [55, 57], whereas one study found more residual PPD for initial shallow pockets in laser monotherapy compared with non-surgical mechanical instrumentation at 6-month follow-up [60, 62]. All the studies reported BoP/BI and PI/PlI; however, 2 articles found statistically significant BoP changes and only 1 study found statistically significant PlI changes between test and control group at 6 months. Mean change in PROMS [59] and GCF biomarker levels/volumes were reported in only 1 article respectively [60], and no significant difference could be found between the treatment modalities at 6 months. For mean changes in subgingival biofilm composition, 4 studies reported relevant results [54, 55, 58, 60, 61]. Two studies failed to distinguish any difference from microorganisms in the periodontal pockets between Er:YAG laser and SRP groups 6 months, 1 year, and 2 years after treatment [54, 55, 58]. In one study, laser therapy yielded statistically significantly lower total bacterial loads (TBL) at 6 months compared with conventional SRP treatment [61]. On the contrary, the detection rate of *Porphyromonas gingivalis* (*Pg*) in the Er:YAG laser group at 6 months was higher than in SRP group in one study [60].

**Table 4:** Summary of selected data with laser monotherapy compared with non-surgical mechanical instrumentation alone

Article (year)	Mean PPD change	Residual PPD	Mean CAL change	Mean BoP/BI change	Mean PI/PLI change	Mean changes in subgingival biofilm composition	Mean changes in GCF biomarker levels	PROMS
Schwarz (2001 and 2003)	+,+*	+,+*	+,+*	+,+*	−	−	NR	NR
Sculean (2004)	−	NR	−	−	−	NR	**NR**	NR
Crespi (2007)	+*	+*	+*	−	−	NR	NR	NR
Kamma (2009)	−	−	−	−	−	NR	NR	NR
Rotundo (2010)	−	−	−	−	−	NR	NR	−
Wang (2017)	−	+^1^	−	−	−	+	−	NR
Grezch (2018)	−	-	+^1^	+	+	+	NR	NR
Total (statistically significant)	2	3	3	2	1	4	**0**	**0**

^1^For shallow sites (1–3 mm); ^2^for moderately deep sites (4–6 mm); ^3^for deep sites (> 7 mm); +statistically significant, −statistically not significant, *1-year outcome; 2-year outcomes, *NR* not reported, *NA* not applicable

### Quality assessment

The assessment of risk of bias of the included studies is illustrated in Table 5 and was based on the Cochrane Center and CONSORT guidelines (Consolidated Standards of Reporting Trials) to evaluate the quality of RCTs [21, 63].

**Table 5:**
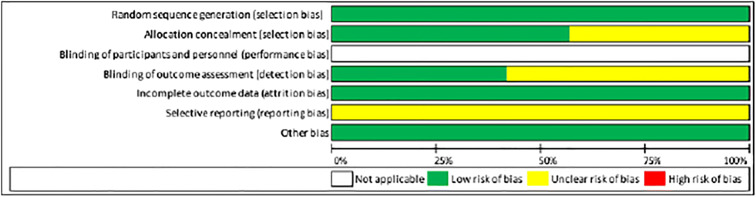

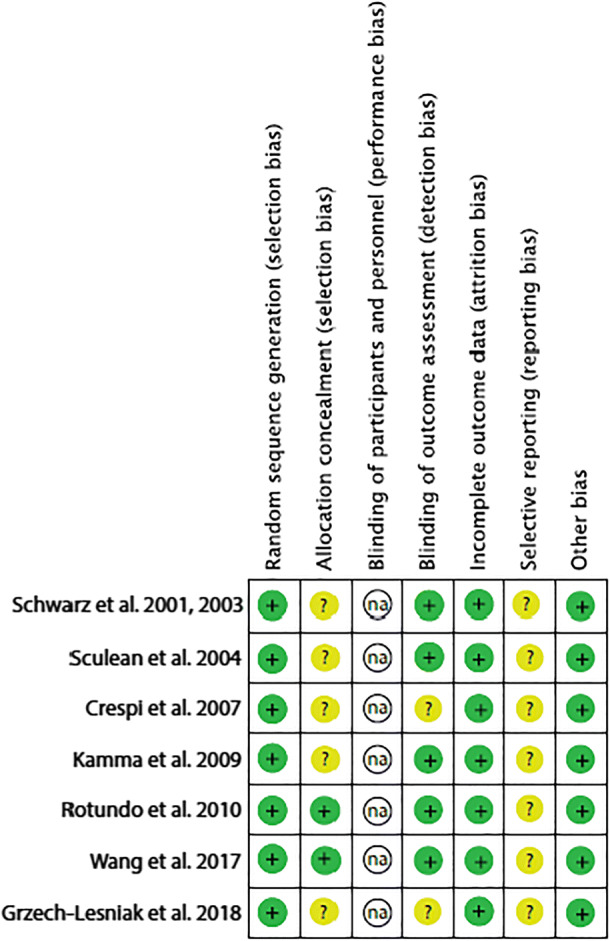
Parameters to evaluate the quality of randomized controlled trials (RCT) according to the Cochrane Center and CONSORT guidelines

## Discussion

The aim of the present systematic review was to investigate the effects of laser application as monotherapy of at least 20 patients with untreated periodontitis after a follow-up of 6 months and to compare them with non-surgical mechanical instrumentation alone. It should be noted that studies reporting on adjunctive laser application to conventional mechanical instrumentation procedures and studies conducted in treated periodontitis patients and in patients enrolled in supportive periodontal therapy were not considered for the present systematic review. The results indicated that in untreated periodontitis patients, laser monotherapy failed to yield superior clinical benefits compared with non-surgical mechanical instrumentation alone.

The purpose of using laser for periodontal therapy is based on the premise that lasers are effective for subgingival calculus removal [13, 64], for reduction of inflammatory mediators including interleukin (IL)-1β or tumor necrosis factor (TNF)-α [65], and for reduction of subgingival bacterial deposits [66]. On the other hand, the concern of using lasers to treat periodontitis is the potential damage of the root surface, the bone, and the surrounding tissues. Because of its emission wavelength (2.94 μm) which is highly absorbed in water and hydroxyapatite and its thermomechanical ablation mechanism, the Er:YAG laser is considered the most effective laser in periodontal treatments [12] removing subgingival calculus while leading to minimal thermal damage to adjacent periodontal tissues and without affecting the dental pulp [67–69]. Other lasers such as CO_2_ and Nd:YAG lasers which are commonly used as high power lasers are not suitable for ablation of hard tissues such as the root surface or the alveolar bone as they carbonize these tissues and exert severe thermal side effects on the target and surrounding tissues [19]. Rather, they are effective for soft tissue surgery. Thermal injury is a major concern when applying laser irradiation. Ablation by means of Er:YAG laser has been reported to induce a temperature rise of less than 6 °C directly on the root surface and of 0.6–2.2 °C in the pulpal wall [19] which does not cause damage to the pulp. However, one histological study, which compared the effects of the Er:YAG laser instrumentation of diseased root surfaces and mechanical removal of plaque and subgingival calculus with SRP, showed that ablation by means of laser resulted in an increased loss of cementum and roughened tooth surfaces [18]. Compared with laser treatments, Gomez et al. (2009) found ultrasonic instrumentation to better preserve the original morphology and microstructure of root cementum [41]. Concerning bone damage, it has been shown that laser-mediated cutting with an Er:YAG laser preserved the trabecular architecture and the thermal and mechanical damages were minimal and limited to the margins of the cut [70]. By using proper laser tips and selecting ideal energy parameters, possible side effects on the root surfaces caused by the laser irradiation may be reduced [17].

The outcomes of two meta-analyses of the present systematic review revealed an additional benefit of 0.14 mm PPD change and of 0.08 mm CAL change in favor of the Er:YAG laser compared with non-surgical instrumentation alone. These results are in line with earlier reviews [13, 71] showing that Er:YAG monotherapy yielded similar clinical results as conventional mechanical debridement. Our review complements the existing reviews, however focused on RCTs with a follow-up of at least 6 months and only included studies reporting on clinical data. Both procedures resulted in significant PPD reductions with initially deeper pockets presenting the greatest reduction [55]. When correlating probing depths and clinical results, 5 studies looked at initial probing depths. Shallow pockets showed a higher CAL loss when non-surgical mechanical instrumentation alone was performed compared with laser treatment [61]. For medium and deep pockets, both treatments yielded similar CAL gain [61]. Interestingly, Crespi et al. (2007) and Wang et al. (2017) found a greater benefit for Er:YAG laser in deeper pockets and inaccessible sites compared with non-surgical mechanical instrumentation alone. One of the major limitation of conventional SRP treatment is the difficult access in deep pockets, furcation areas, and root concavities [72]. Rabbani et al. (1981) showed a high correlation between pocket depth and the amount of residual calculus: the deeper the pocket, the more difficult to remove the calculus thoroughly by hand instruments [73]. Conversely, due to the small size of the laser tip, lasers have the advantage of treating otherwise inaccessible areas and sites. Ge et al. (2017) evaluated the clinical use of Er,Cr:YSGG laser in the management of degree II or III furcation involvement, and their results demonstrated that the reduction of PPD and BOP after 6 and 12 months was higher in the laser group than in the conventional root planing group [39].

An early study compared the effectiveness of subgingival calculus removal by Er:YAG laser to hand instrumentation. 83.3 ± 5.7% of the root surface was calculus-free after laser irradiation in contrast to 93.9 ± 3.7% after SRP with half of the treatment time used for laser therapy [11]. In terms of time efficiency, others reported a lower efficiency for ablation by means of Er:YAG laser when compared with conventional ultrasonic scaling [19]. Also in this study, it was shown that Er:YAG laser–treated surfaces were macroscopically rougher or most similar to ultrasonically scaled roots [19]. In combination with a fluorescent calculus detection system, Er:YAG laser enabled equal or even more effective removal of subgingival calculus and a predictable preservation of root surface structure compared with SRP [74, 75]. The question then arises whether or not Er:YAG laser therapy is an additional tool in conjunction with open flap debridement (OFD)[76]. It should be noted that laser acquisition entails substantial costs, yet data reporting on cost-effectiveness are still missing.

This review has some limitations. First, not many RCTs present data of over 2 years, here only 2 studies [55, 57] report on 2-year data. Second, only one of the included studies reported on residual PPD > 5 mm although, from a clinical perspective, residual PPD are the main parameter to evaluate therapeutic success and further treatment needs. Third, no study reported on time effectiveness. Fourth, all but one study were carried out in a split-mouth design with possible carry-over effects.

Taken together, within its limitations, currently available evidence indicates that in patients with untreated periodontitis, the single use of Er:YAG laser does not seem to yield clinical advantages over non-surgical mechanical instrumentation alone in terms of PPD and CAL changes.
